# P2X7 Receptor Induces Tumor Necrosis Factor-α Converting Enzyme Activation and Release to Boost TNF-α Production

**DOI:** 10.3389/fimmu.2017.00862

**Published:** 2017-07-24

**Authors:** Maria Barberà-Cremades, Ana I. Gómez, Alberto Baroja-Mazo, Laura Martínez-Alarcón, Carlos M. Martínez, Carlos de Torre-Minguela, Pablo Pelegrín

**Affiliations:** ^1^Biomedical Research Institute of Murcia (IMIB-Arrixaca), Clinical University Hospital Virgen de la Arrixaca, Murcia, Spain

**Keywords:** tumor necrosis factor-α, tumor necrosis factor-α converting enzyme, exosomes, macrophages, P2X7 receptor, inflammation, cytokine

## Abstract

Tumor necrosis factor (TNF)-α is a major pro-inflammatory cytokine produced in response to toll-like receptor stimulation. TNF-α release is controlled by the activity of TNF-α converting enzyme (TACE) that cut membrane-bound TNF-α to shed its ectodomain as a soluble cytokine. The purinergic receptor P2X ligand-gated ion channel 7 (P2X7) is activated in response to elevated concentrations of extracellular ATP and induces different pro-inflammatory pathways in macrophages to establish an inflammatory response. P2X7 receptor promotes the activation of the inflammasome and the release of interleukin-1β, the production of inflammatory lipids, and the generation of reactive oxygen species. In this study, we analyzed the mechanism of P2X7 receptor responsible of TNF-α release after priming macrophages with LPS doses ≤100 ng/ml. We found that P2X7 receptor increases the extracellular activity of TACE through the release of the mature form of TACE in exosomes. This effect was blocked using P2X7 receptor inhibitors or in macrophages obtained from P2X7 receptor-deficient mice. Elevation of intracellular Ca^2+^ and p38 mitogen-activated protein kinase after P2X7 receptor activation were involved in the release of TACE, which was able to process TNF-α on nearby expressing cells. Finally, we observed an increase of TNF-α in the peritoneal lavage of mice treated with LPS and ATP. In conclusion, P2X7 receptor induces the release of TACE in exosomes to the extracellular compartment that could amplify the pro-inflammatory signal associated to this receptor. These results are important for the development of therapeutics targeting P2X7 receptor.

## Introduction

The coordinated response of the innate immune system observed during inflammation requires the activation of a complex system of sensors to induce the release of different signaling molecules (1). The purinergic receptor P2X ligand-gated ion channel 7 (P2X7) is one of these sensors responsible to identify elevated concentrations of the extracellular nucleotide ATP, considered a danger signal at sites of tissue damage (2). The activation of P2X7 receptor by extracellular ATP is a well-known physiological trigger of the NLRP3 inflammasome and the release of the pro-inflammatory cytokine interleukin (IL)-1β (3). IL-1β release follows an unconventional protein release pathway independent on the endoplasmic reticulum and dependent on the permeabilization of the plasma membrane (4). IL-1β is a key cytokine implicated in the development of different chronic inflammatory diseases and, therefore, P2X7 receptor is a potential target to develop anti-inflammatory drugs to treat chronic inflammatory conditions as rheumatoid arthritis or Crohn’s disease (5–7). Mice lacking P2X7 receptor present reduced joint destruction in models of arthritis and delayed hypersensitivity in inflammatory bowel disease (8, 9). Beyond IL-1β release, P2X7 receptor stimulation in macrophages also lead to the production of reactive oxygen species (ROS) and inflammatory lipids (10, 11), leading to the establishment of a pro-inflammatory environment. Recently, we have characterized the proteins released from macrophages after P2X7 receptor stimulation, such secretome included the cytokine tumor necrosis factor (TNF)-α (12). TNF-α is a master cytokine for the inflammatory response, contributing to different inflammatory conditions, and therapeutics blocking TNF-α signaling are approved drugs for the treatment of rheumatoid arthritis or Crohn’s disease (13, 14). TNF-α production is induced by toll-like receptor that increases *TNFA* gene transcription and the translation of TNF-α as an integral membrane protein that traffics from the endoplasmic reticulum to the plasma membrane. The ectodomain of the plasma membrane-bound TNF-α is then cut by the action of TNF-α converting enzyme (TACE) to release the soluble form of this cytokine (15). TACE is a plasma membrane member of the ADAM family of metalloproteases (a disintegrin and metalloprotease; ADAM-17), which activity is modulated by mitogen-activated protein kinases (MAPKs) and ROS (16).

In this study, we explore the role of P2X7 receptor inducing the release of TACE and TNF-α *via* intracellular Ca^2+^ increase and p38 MAPK activity. We found that extracellular TACE was present in exosomes produced upon P2X7 receptor stimulation.

## Materials and Methods

### Cells, Reagents, and Buffers

Key reagents and their sources were as follows: *Escherichia coli* LPS serotype 055:B5, TNF-α protease inhibitor-0 (TAPI-0) and ATP were from Sigma-Aldrich; selective p38 inhibitor (SB202190) was from Calbiochem Merck-Millipore; P2X7 receptor selective antagonists AZ10606120, A438079, and A740003 were from Tocris. The composition of the physiological buffer used in all experiments to stimulate macrophages with ATP was (in millimoles): 147 NaCl, 10 HEPES, 13 d-glucose, 2 KCl, 2 CaCl_2_, and 1 MgCl_2_; pH 7.4.

HEK293T cells (ATCC CRL-11268) were cultured in DMEM:F-12 media (1:1; Lonza) supplemented with 10% of fetal calf serum (Life Technologies) and 2 mM Glutamax (Life Technologies) and were routinely tested for mycoplasma contamination with a Mycoplasma Detection Kit (Roche). Lipofectamine 2000 (Life Technologies) was used according to the manufacturer’s instructions to transfect a plasmid coding for human TNF-α into HEK293T cells.

### Human Samples

Whole peripheral blood samples were collected from healthy donors upon approval of the *Hospital Clínico Universitario Virgen de la Arrixaca*’s Clinical Review Board. An informed consent was obtained from all donors enrolled in the study following the principles set out in the WMA Declaration of Helsinki. Human peripheral blood mononuclear cells were isolated following standard procedure (10) and cultured for 16 h in RPMI 1640 medium (Lonza) with 10% of FCS, 2 mM Glutamax, and 100 U/ml penicillin–streptomycin (Life Technologies). After monocyte adherence, cells were washed and primed for 4 h with LPS (10 ng/ml), and then cells were washed or not with physiological buffer and incubated in the same buffer at 37°C with 3 mM of ATP for 20 min.

### Mice

P2X7 receptor-deficient mice in C57BL/6 background (*P2rx7*^−/−^) (17) were purchased from Jackson. For all experiments, mice between 8 and 10 weeks of age bred under SPF conditions were used in accordance with the *Hospital Clínico Universitario Virgen Arrixaca* animal experimentation guidelines, and the Spanish national (RD 1201/2005 and Law 32/2007) and EU (86/609/EEC and 2010/63/EU) legislation. Animal procedure was refined and approved by the *Hospital Clínico Universitario Virgen Arrixaca* animal experimentation committee and approved by the *Servicio de Sanidad Animal, Dirección General de Ganadería y Pesca, Consejería de Agricultura y Agua Región de Murcia* (Health Animal Service, Murcia Fishing and Farming Council, reference C1310050308). C57BL/6 [wild-type (WT)] and *P2rx7*^−/−^ mice were injected i.p. with 200 µl of LPS (50 µg/kg) in sterile phosphate-buffered solution (PBS). Two hours after this LPS injection, mice were injected i.p. with either 0.5 ml of ATP (1.5 M/kg) or PBS. Mice were euthanized by CO_2_ inhalation 120 min after ATP or PBS injection, and each peritoneal cavity was washed with 3 ml of PBS. Individual lavages were centrifuged, supernatants were collected, and cellular pellet was used to detect the number of peritoneal Gr-1/Mac1^+^ cells by flow cytometry in a FACScalibur flow cytometer (Beckton-Dickinson Biosciences) as described elsewhere (18).

### Differentiation of Macrophages from Mouse Bone Marrow Precursors and *In Vitro* Stimulation

Bone marrow-derived macrophages (BMDMs) were obtained as described (10). After differentiation, BMDMs were plated at a confluence of 0.42 × 10^6^ cells/cm^2^ in 24-well plates. The day after seeding, macrophages were stimulated with LPS (if not indicated otherwise, 4 h at 10 ng/ml). Cells were then washed twice for pulse-chase experiments with physiological buffer and if not indicated otherwise incubated in the same buffer for 20 min with ATP at 3 mM. In accumulative experiments, macrophages were stimulated with ATP on the top of the LPS priming medium without washing the LPS. In other experiments as explained in the figure legends, BMDMs were pretreated with various pharmacological compounds 10 min before and during ATP stimulation. After ATP treatment, supernatants were collected and clarified at 14,000 *g* for 30 s at 4°C to remove floating cells and stored at −80°C until cytokine determination.

### Purification of Exosomes

Exosomes purification was performed as previously described (19). Briefly, differentiated BMDMs in 150 mm^2^ plates were washed with PBS and incubated 24 h in medium with exosomes-depleted FBS. The cells were primed with 10 ng/ml LPS for 4 h at 37°C, followed by washing three times with physiological buffer and incubated in the same buffer for 20 min with ATP at 3 mM. The collected medium was immediately transferred into a tube containing Protease inhibitors mix (Sigma) on ice and then followed by sequential centrifugation at 4°C for 20 min at 2,000 *g* (Sigma 3-18KS, rotor 11180&13190), 30 min at 10,000 *g*, and 1 h at 100,000 *g* (Beckman Ultracentrifuge Optima L-80 XP, SW40 rotor). The supernatant of this last step was named as S100 and was stored at −80°C. The pellet from 100,000 *g* was washed in 10 ml of PBS and centrifuged again for 1 h at 100,000 *g*. Finally, exosomal fraction was collected in the pellet with 50 µl of PBS and stored at −80°C until their use.

### Transmission Electron Microscopy

Electron microscopy analysis was performed as previously described by Théry et al. (19), on pellets of purified exosomes loaded on Formvar–carbon-coated grids and fixed in 2% paraformaldehyde. Grids were observed at 80 kV with a JEM-1011 Transmission Electron Microscope (JEOL Company).

### Western Blots

Cells lysates, precipitated cell-free supernatants, exosomes preparation, and precipitated S100 supernatants were resolved in 4–12% polyacrylamide gels and electrotransferred as it is described in de Torre-Minguela et al. (12). Membranes were probed with different antibodies: anti-ADAM17 (TACE) rabbit polyclonal (ab2051, Abcam), anti-CD9 rabbit monoclonal (EPR2949, ab92726, Abcam), and horseradish peroxidize-anti-β-actin (C4; sc-47778HRP, Santa Cruz).

### ELISA Assays

Individual mouse peritoneal lavages or culture cell-free supernatants were collected, clarified by centrifugation and the concentration of IL-1β and TNF-α was tested by the mouse or human ELISA kit following manufacturer’s instructions (R&D Systems).

### TACE Activity

TNF-α converting enzyme activity was measured in macrophage lysates or cell-free supernatants using the SensoLyte^®^ 520 TACE (α-secretase) fluorimetric activity kit (AnaSpec) following the manufacturer’s instructions. Briefly, cell lysates and undiluted cell-free supernatants from 2 × 10^6^ macrophages were diluted in assay buffer with 1 µM of the FRET substrate LAQAVRSSSR labeled with 5-carboxyfluorescein fluorophore quenched by QXL™520. After 30 s shaking to mix the components, TACE activity was measured using a black clear bottom 96-well plate (Costar Corning Life Sciences) in a Synergy Mx (Biotek) plate reader, at excitation = 490 ± 6.75 nm and emission = 520 ± 6.75 nm every 5 min during 40 min at 30°C. The values used were the fluorescence intensity at 20 min and values shown in results were expressed as relative fluorescence units (rfu).

### Statistical Analysis

**Figure 1:**
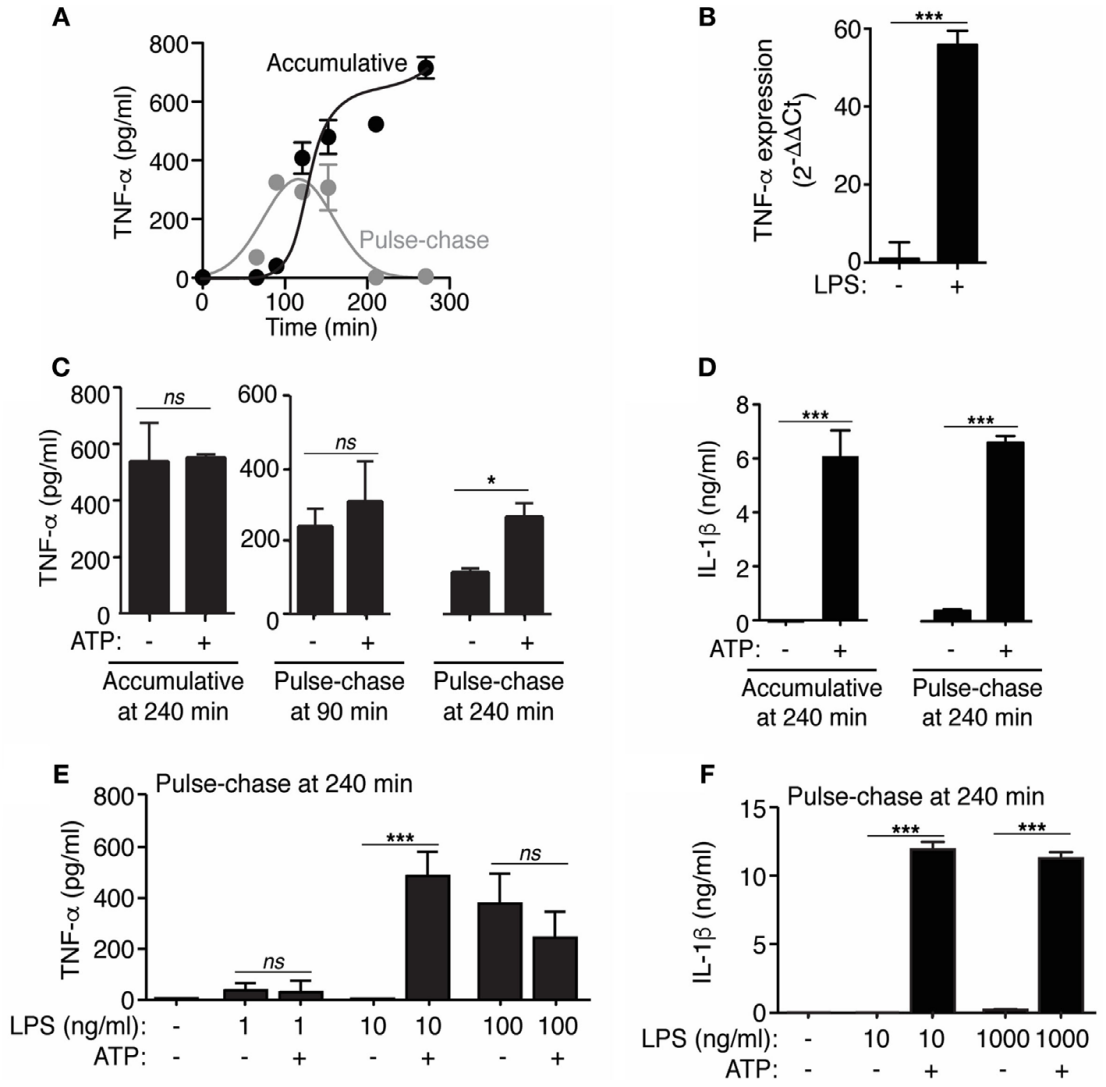
Extracellular ATP modulates tumor necrosis factor-α (TNF-α) release. **(A)** ELISA for TNF-α in bone marrow-derived macrophages (BMDMs) supernatants after stimulation with 10 ng/ml of LPS for different times as indicated (accumulative) or pulsed with LPS for the indicated time and then washed and chased in the absence of LPS for 30 min (pulse-chase); *n* = 2 independent experiments. **(B)** Relative gene expression (2^−ΔΔCt^) for TNF-α determined by quantitative RT-PCR from BMDMs unprimed or primed for 4 h with LPS (1 µg/ml); *n* = 3 independent experiments. **(C,D)** ELISA analysis for released TNF-α **(C)** and interleukin (IL)-1β **(D)** from BMDMs LPS-primed as in **(A)**, but followed by no stimulation or stimulation for 20 min with ATP (3 mM); *n* = 2–6 independent experiments. In panel **(C)**, the error bars represent SD to show the variability obtained in accumulative vs pulse chase experiments. **(E,F)** ELISA analysis for released TNF-α **(E)** or IL-1β **(F)** from BMDMs primed for 4 h with different concentrations of LPS as indicated, followed by no stimulation or stimulation for 20 min with 3 mM of ATP (pulse-chase) **(B)**; *n* = 4 independent experiments.

All data are shown as mean values and error bars represent SE from the number of independent assays indicated in the figure legend, except for Figure 1C that the error bars represent SD to show the variability obtained in accumulative vs pulse chase experiments. For two-group comparisons, Mann–Whitney test was used meanwhile comparisons of multiple groups were analyzed by Kruskal–Wallis test using Prism software (Graph-Pad Software, Inc.). *p-*Value is indicated as ***p < 0.001; **p > 0.001 < 0.01; *p > 0.01 < 0.05; *p* > 0.05 not significant (ns).

## Results

### TNF-α Release Decreases with the Time of LPS Stimulation

LPS induced the accumulation of TNF-α in the supernatant of macrophages with an exponential increase from 90 to 120 min and then reached a plateau (Figure 1A), suggesting a decrease on TNF-α release with the time. This decline on TNF-α release was evident when the cytokine was detected in “pulse-chase” experiments, where the cells were “pulsed” with LPS for different times, washed to remove accumulated TNF-α in the medium and the new release of TNF-α was “chased” for 30 min in fresh medium without LPS (Figure 1A). In these experimental conditions, we found a decrease on TNF-α release after 150 min of LPS stimulation (Figure 1A), and this decline was not due to a lack of *Tnfa* gene expression (Figure 1B) or to an intracellular deficiency of TNF-α, since stimulation with 3 mM of ATP in pulse-chase experiments after 240 min of LPS treatment was able to increase TNF-α concentration on cell supernatants (Figure 1C). This result is in line with our recent work identifying TNF-α as one of the proteins released in LPS-primed macrophages after the activation of P2X7 receptor by extracellular ATP (12). Cell stimulation with ATP did not affect extracellular TNF-α levels in accumulative assays or pulse-chase experiments when TNF-α release reached its maximum level (90 min) (Figure 1C), this could be due to saturation of TNF-α detection on supernatants after LPS stimulation. However, IL-1β release was strongly induced by ATP in both accumulative and pulse-chase experiments (Figure 1D). Similar results were found when different doses of LPS were used to prime macrophages and ATP was unable to increase TNF-α release in pulse-chase experiments when LPS was used at a dose >100 ng/ml in contrast to IL-1β release (Figures 1E,F). These data suggest a pro-inflammatory role of P2X7 receptor beyond IL-1β when macrophages are exposed to low levels of LPS.

### P2X7 Receptor Differentially Controls TNF-α and IL-1β Release in Macrophages

**Figure 2:**
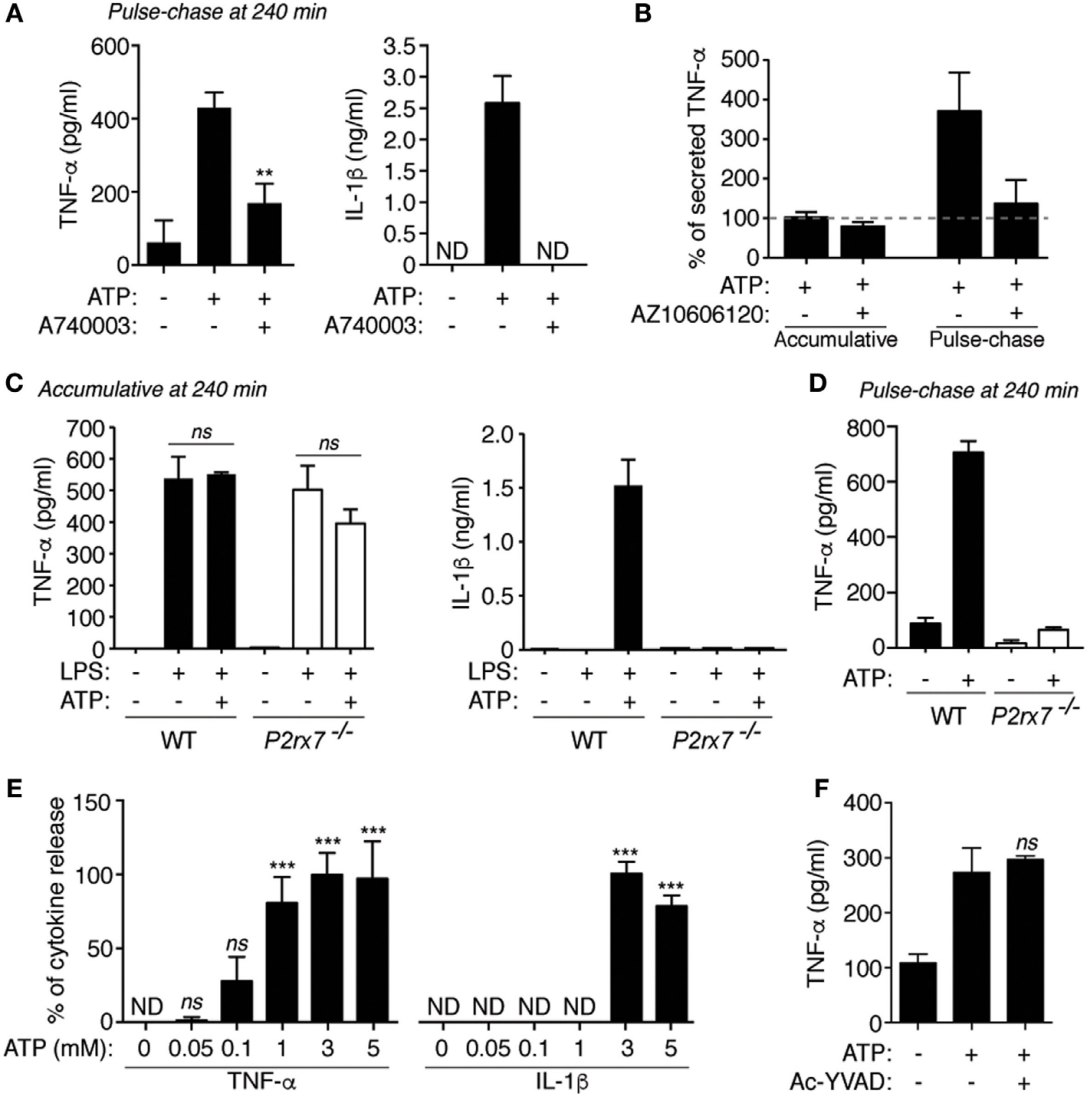
P2X7 receptor differentially controls tumor necrosis factor-α (TNF-α) and interleukin (IL)-1β release. **(A,B)** ELISA analysis for released TNF-α **(A,B)** or IL-1β **(A)** from BMDM **(A)** or from human blood mononuclear cells isolated from healthy donors **(B)** primed for 4 h with LPS, then washed [**(A)**, pulse-chase in **(B)**] or not [accumulative in **(B)**] and stimulated with 20 min of ATP; 10 min before and during ATP stimulation cells were incubated with the specific P2X7 receptor inhibitor (A740003 or AZ10606120, both at 10 µM); *n* = 4–5 independent experiments **(A)** or *n* = 4 healthy donors for the accumulative experiments and *n* = 6 healthy donors for the pulse-chase experiment **(B)**. The average concentration of TNF-α released in accumulative experiments was 203.7 pg/ml and it was considered as 100% in panel **(B)**. **(C,D)** ELISA analysis for released TNF-α **(C,D)** or IL-1β **(C)** from wild-type (WT) or P2X7 receptor-deficient (*P2rx7*^−/−^) BMDM primed for 4 h with LPS, then washed [**(D)**, pulse-chase] or not [**(C)**, accumulative] and stimulated with 20 min of ATP; *n* = 2–3 independent experiments. **(E,F)** ELISA analysis for released TNF-α **(E,F)** or IL-1β **(E)** from BMDM primed for 4 h with LPS, then washed (pulse-chase), and stimulated with 20 min of ATP [different concentrations in **(E)**, or 3 mM in **(F)**]; 10 min before and during ATP stimulation cells were incubated with the caspase-1 inhibitor Ac-YVAD (100 µM), **(F)**; *n* = 4 independent experiments **(E)** or *n* = 3 **(F)**.

The specific P2X7 receptor antagonist A740003 was able to decrease the release of TNF-α and IL-1β induced by ATP in pulse chase experiments (Figure 2A). Similarly, in human peripheral blood mononuclear cells, the release of TNF-α was reduced in pulse chase experiments, but not in accumulative experiments, when ATP treatment was applied in combination with the P2X7 receptor blocker AZ10606120 (Figure 2B). Consistently, *P2rx7*^−/−^ macrophages present release of TNF-α when ATP was added in accumulative assays (Figure 2C), but it was affected when ATP stimulation was used in pulse-chase experiments (Figure 2D). The release of IL-1β, which is highly dependent on P2X7 receptor activating caspase-1, was affected in *P2rx7*^−/−^ macrophages in accumulative assays (Figure 2C). However, meanwhile the release of IL-1β was only detected using concentrations of ATP higher than 3 mM, TNF-α release increased at concentrations of 1 mM ATP (Figure 2E). Interestingly, we then found that P2X7 receptor-induced TNF-α release was not impaired by using a caspase-1 inhibitor (Figure 2F), confirming our previous results where ATP-induced TNF-α release was independent of the NLRP3 inflammasome pathway (12).

**Figure 3:**
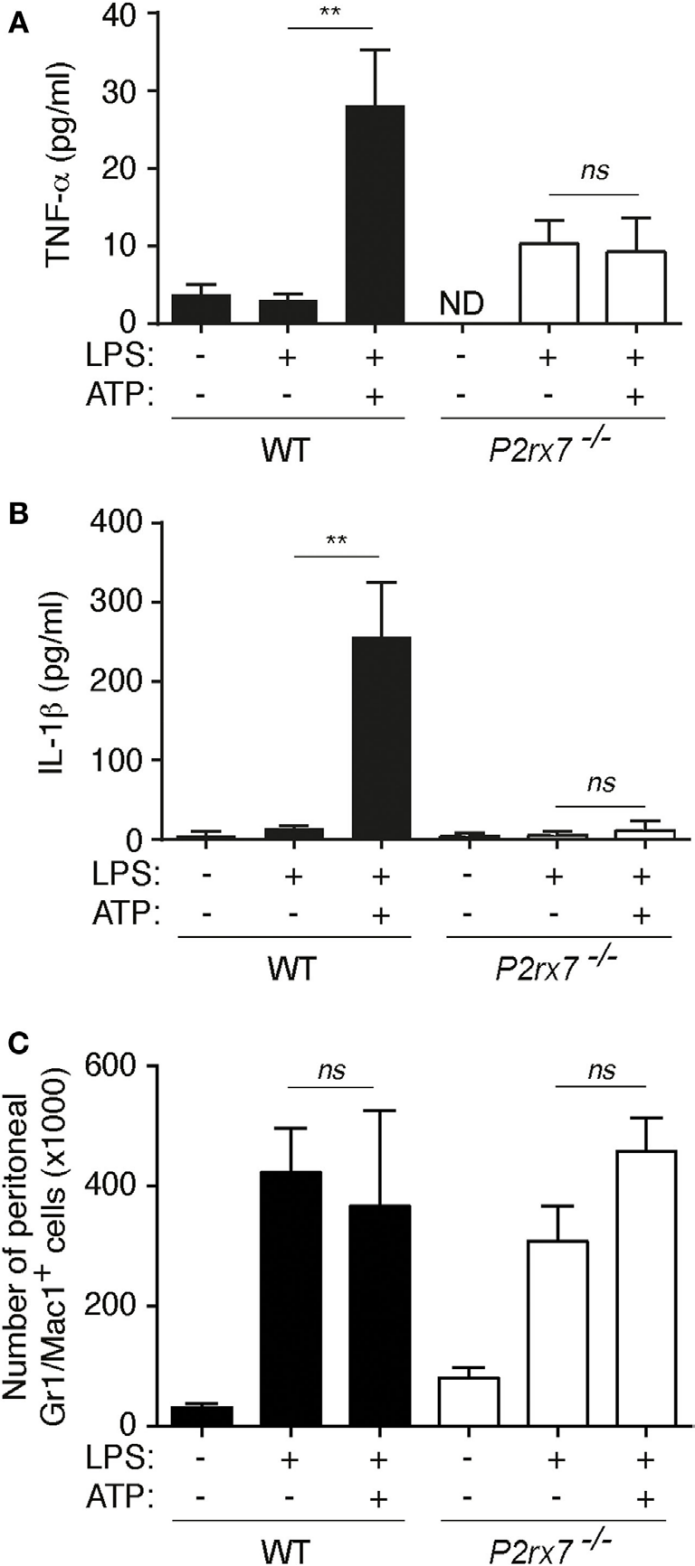
P2X7 receptor induces tumor necrosis factor-α (TNF-α) release *in vivo*. **(A–C)** Peritoneal TNF-α **(A)**, interleukin (IL)-1β **(B)**, or number of Gr-1/Mac-1 double-positive cells **(C)** in wild-type (WT) or P2X7 receptor-deficient (*P2rx7*^−/−^) mice 2 h after intraperitoneal injection of vehicle or 50 µg/kg of LPS followed by a second intraperitoneal injection of vehicle or 1.5 M/kg ATP for 2 h; *n* = 3–9 animals per group.

As an *in vivo* proof of concept, intraperitoneal challenge of mice with LPS and ATP resulted in an increase of peritoneal TNF-α when compared to LPS injected animals, and as control, IL-1β was also increased upon LPS and ATP injection (Figures 3A,B). In *P2rx7*^−/−^ mice, there was no potentiation of TNF-α and IL-1β by ATP (Figures 3A,B). ATP treatment did not change the increase of infiltrated peritoneal Gr-1/Mac-1-positive cells (Figure 3C). This result contrast with previous publications showing that ATP increases granulocyte infiltration to the peritoneum (20, 21); however, in such studies, ATP was used at lower concentration and without endotoxin that might explain the differences.

### P2X7 Receptor Stimulation Controls TACE Activity in Macrophages

**Figure 4:**
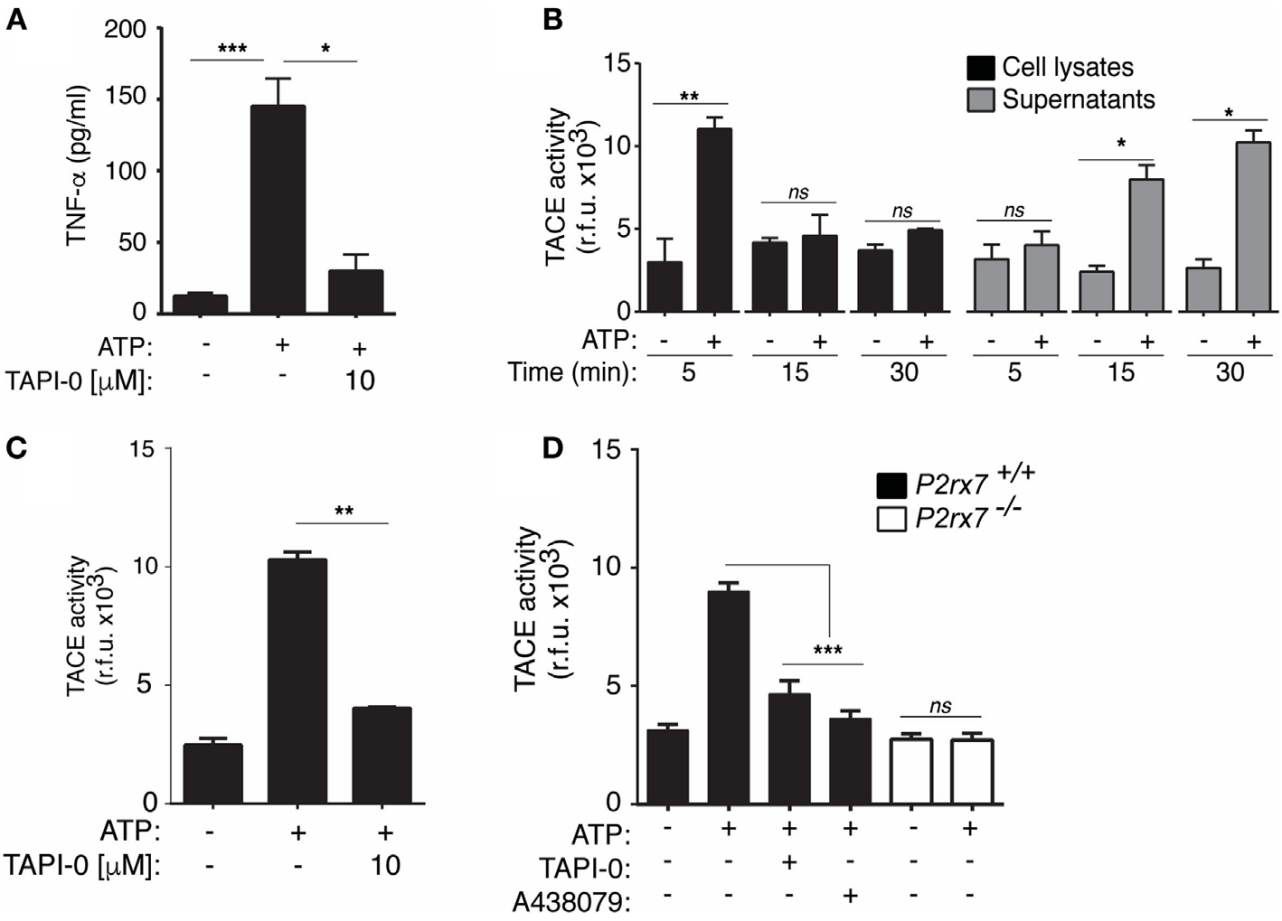
P2X7 receptor stimulation controls TNF-α converting enzyme (TACE) activity and release in macrophages. (**A)** ELISA analysis for released tumor necrosis factor-α (TNF-α) from BMDM primed for 4 h with 10 ng/ml LPS, washed, and then stimulated with 20 min of ATP; 10 min before and during ATP stimulation cells were incubated with the specific TACE inhibitor [TNF-α protease inhibitor-0 (TAPI-0), 10 µM]; *n* = 4 independent experiments. **(B–D)** Quantification of TACE activity from wild-type (WT) **(B–D)** or P2X7 receptor-deficient (*P2rx7*^−/−^) **(D)** BMDMs in cell lysates **(B)** or supernatants **(B–D)** primed as in **(A)**, but stimulated with ATP for different times as indicated **(B)** or for 20 min **(C,D)**; additionally, TAPI-0 (10 µM) was added to the supernatants after collecting from the ATP-treated cells and before TACE substrate was added **(C)** or macrophages were incubated for 10 min before and during ATP stimulation **(D)** with the P2X7 receptor antagonist A438079 (25 µM) or TAPI-0 (10 µM); *n* = 3 independent experiments; ND, not detected.

Our results suggest that P2X7 receptor activation could modulate the release of TNF-α, and consistently, the inhibition of the metalloproteinase TACE significantly reduces P2×7 receptor-induced TNF-α release (Figure 4A). We next studied if P2X7 receptor could be modulating TACE activity in macrophages to induce the release of TNF-α. We found an increase of TACE activity in macrophages after 5 min of ATP stimulation and after 15 and 30 min in cell supernatants (Figure 4B), suggesting that TACE was not only activated upon P2X7 receptor stimulation but also released. The stimulation of P2X7 receptor induces the activation and release of several metalloproteases (12, 22) that could process the fluorescence substrate used in the assay as a consequence of their promiscuity. However, TACE activity measured in cell supernatant was blocked when TAPI-0, a widely used TACE inhibitor, was added to the reaction before activity measurement (Figure 4C). Extracellular TACE activity detected after ATP stimulation was reduced in supernatant from macrophages deficient on P2X7 receptor or when the specific P2X7 receptor antagonist A438079 or TAPI-0 was incubated with the cells (Figure 4D).

### P2X7 Receptor-Induced Intracellular Ca^2+^ and MAPK Activation Modulates TACE

**Figure 5:**
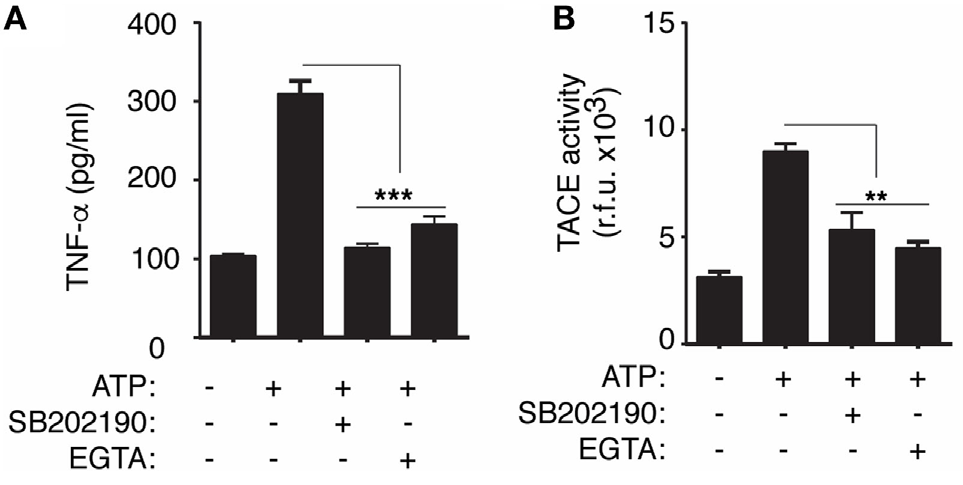
Release of TNF-α converting enzyme (TACE) induced by P2X7 receptor depends on p38 mitogen-activated protein kinase. **(A)** Quantification of TACE activity from BMDMs supernatants primed for 4 h with LPS and then stimulated with 20 min of ATP; 10 min before and during ATP stimulation cells were incubated with p38 inhibitor SB202190 (10 µM) or with Ca^2+^-chelator EGTA (1 mM); *n* = 3 independent experiments. **(B)** ELISA analysis for released tumor necrosis factor-α (TNF-α) from BMDMs activated as in **(A)**, and additionally, 10 min before and during ATP stimulation cells were incubated with SB202190 (10 µM) or EGTA (1 mM); data are presented as mean and SEM of *n* = 4 independent experiments.

Since P2X7 receptor activates MAPK *via* intracellular Ca^2+^ rise and p38 MAPK controls activation of TACE (16, 23), we next wondered if P2X7 receptor could be modulating TACE activity. We found that upon P2X7 receptor activation, extracellular TACE activity was reduced when macrophages were treated with the p38 inhibitor SB202190 or when intracellular Ca^2+^ rise was prevented using an extracellular medium with no Ca^2+^ and supplemented with EGTA (Figure 5A). In parallel, the release of TNF-α induced by ATP treatment was also significantly reduced after treatment with SB202190 or preventing intracellular Ca^2+^ rise (Figure 5B).

### P2X7 Receptor Induces the Release of Biological Active TACE in Exosomes

**Figure 6:**
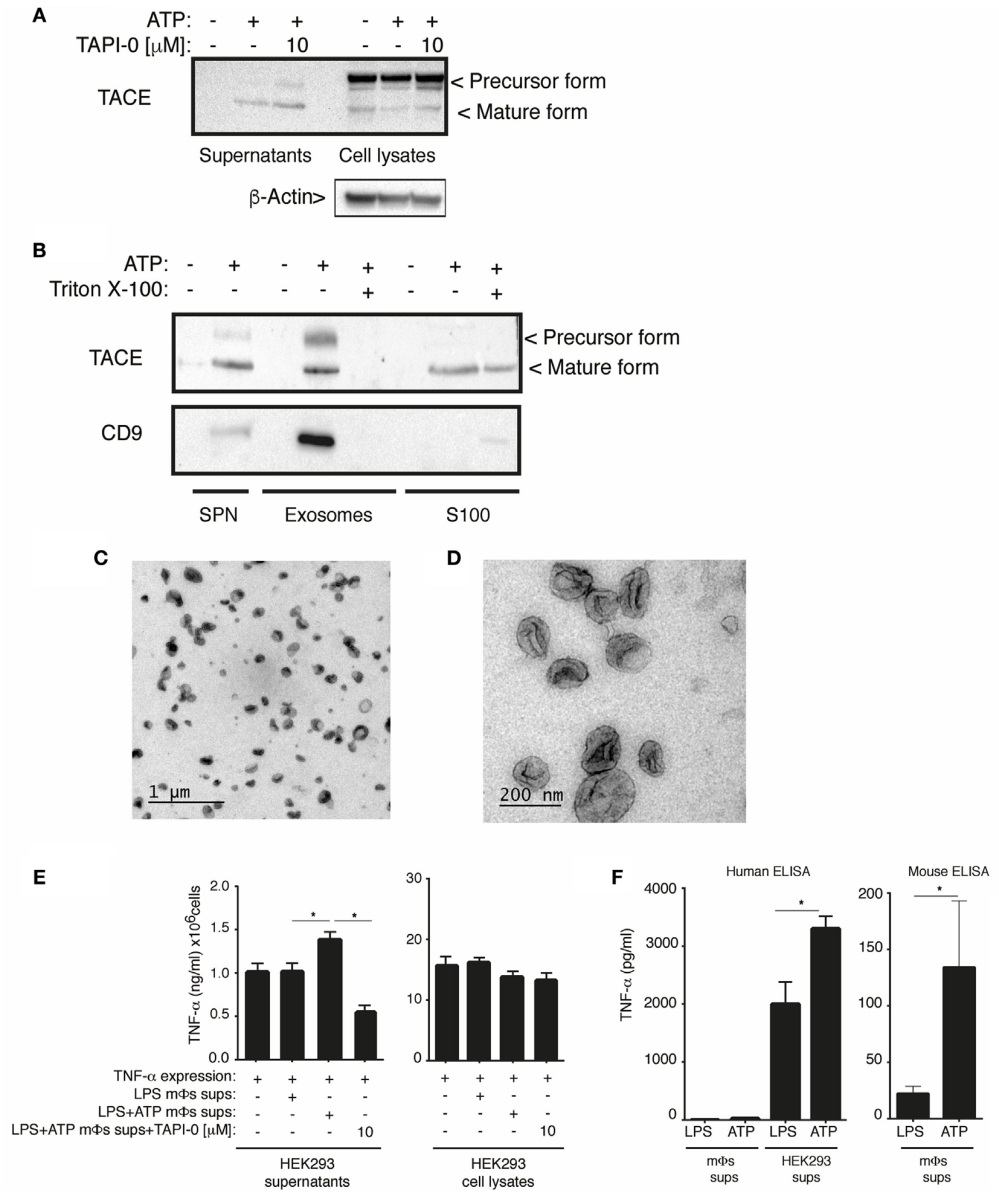
Active form of TNF-α converting enzyme (TACE) is release in exosomes upon P2X7 receptor activation. **(A)** Western blot analysis of cell-free supernatants and cell lysates from BMDM primed for 4 h with 10 ng/ml LPS, washed, and then stimulated with 3 mM of ATP 20 min; 10 min before and during ATP stimulation cells were incubated with the TACE inhibitor (TAPI-0, 10 µM). Images are representative of *n* = 3 independent experiments, **(B)** western blot analysis of cell-free supernatants, exosomal, and soluble fraction (S100) obtained from BMDM primed as in **(A)**. In indicated lanes, the cell-free supernatant was treated with 20% of Triton X100 (30 min) before the exosomes purification. Images are representative of *n* = 5 independent experiments. **(C,D)** Representative transmission electron microscopy images of exosomal fraction obtained from cell-free supernatant of BMDM primed as in **(A)** and stimulated with 3 mM of ATP 20 min. Scale bar: 1 µm **(C)** or 200 nm **(D)**. **(E)** Released and cell-associated human tumor necrosis factor (TNF)-α detected by ELISA from HEK293T cells expressing human TNF-α and incubated during 30 min with supernatants from BMDMs primed for 4 h with 10 ng/ml LPS, washed, and then stimulated with 20 min of ATP. Five minutes before stimulation of the cells, ATP supernatant were incubated with TAPI-0 (10 µM); *n* = 4 independent experiments. Mϕs: macrophages. **(F)** Quantification of TNF-α released in cell-free supernatant of BMDM and HEK293 cells described in **(E)** using ELISA kit against human or mouse TNF-α as indicated; *n* = 4 independent experiments.

TNF-α converting enzyme is a type-I transmembrane protease synthesized as an inactive precursor that requires a maturation process in the Golgi apparatus, where the Furin convertase removes the prodomain of the zymogen to activate it (24). The precursor and mature forms of TACE were detected in cell-free supernatants obtained from macrophages after P2X7 receptor stimulation. The release of TACE was not reduced by TAPI-0 (Figure 6A), although this inhibitor reduced the release of TNF-α (Figure 4A), suggesting that the release of TACE is independent on its activity.

The extracellular TACE activity induced after P2X7 receptor activation could be associated with extracellular vesicles since P2X7 receptor induces the release of extracellular vesicles from macrophages (25) and TACE is a membrane-bound enzyme. Interestingly, after ATP stimulation, the two forms of TACE were detected in the exosomal fraction of supernatants and only the mature form of TACE was detected in the supernatant soluble fraction (S100), obtained after exosomes removal (Figure 6B). The detection of the membrane exosome marker CD9 (26) and the size of the vesicles (20–100 nm) purified in exosomal fraction (Figures 6C,D) suggest that, after P2X7 receptor activation, TACE is released in exosomes. Furthermore, the treatment of whole cell-free supernatant with Triton X100 before exosomes purification to disintegrate these vesicles before the fractionation resulted in a loose of TACE and CD9 from the exosomal fraction (Figure 6B). The antibody used in these experiments to detect TACE was generated against an epitope of the C-terminal of this protein. Therefore, based on the molecular size observed, the mature form of TACE detected in the soluble fraction (S100) could be a product of vesicles disintegration during the fractionation protocol.

We then aimed to analyze if released TACE after P2X7 receptor stimulation could have a physiological role inducing the shedding of plasma membrane-bound TNF-α from neighboring cells. We expressed human TNF-α in HEK293T cells and incubated them with cell-free supernatants from macrophages activated or not with ATP and found that cell-free supernatants from ATP-treated mouse macrophages were able to significantly increase the release of human TNF-α from HEK293T cells (Figure 6E). In these assays, mouse TNF-α released by the macrophages had not influenced in the increase of human TNF-α detected by ELISA although we tested that ATP was potentiating the release of mouse TNF-α in these macrophages samples (Figure 6F). Moreover, the addition of the TACE inhibitor TAPI-0 to the cell-free supernatant from ATP-treated mouse macrophages reduced the release of TNF-α from HEK293T expressing TNF-α (Figure 6E), suggesting that released TACE from P2X7 receptor-activated macrophages was able to shed TNF-α from neighborhood cells.

In summary, our data present P2X7 receptor as a regulator of TACE activity that potentiates TNF-α release in macrophages when cytokine release decreases upon LPS stimulation. In addition, the release of the active TACE in exosomes after activation of the P2X7 receptor can shed membrane proteins present in neighboring cells, such as TNF-α, acting as a mechanism for the propagation of the inflammation.

## Discussion

There are two main findings from the present study: first, we have identified TACE activation and TNF-α release associated to P2X7 receptor in macrophages, and second, P2X7 receptor signaling induces the release of mature TACE in exosomes that could shed membrane proteins from neighboring cells, being a potential mechanism to spread the inflammatory signaling.

P2X7 receptor is an ionotropic channel gated by high concentrations of extracellular ATP, its activation leads to the opening of a cationic non-specific pore that allows Ca^2+^ entry to the cell and intracellular K^+^ efflux (27, 28). In LPS-primed macrophages, P2X7 receptor K^+^ efflux leads to the activation of the NLRP3 inflammasome and the subsequent release of IL-1β (29, 30). Besides, P2X7 receptor Ca^2+^ influx mediates the activation of phospholipase A and the production of inflammatory lipids as prostaglandins or thromboxans (10, 31). Intracellular Ca^2+^ rise induced by P2X7 receptor also modulates the generation of ROS (32, 33), being ROS generation important for P2X7 receptor-induced NLRP3 inflammasome activation (34, 35). Our results demonstrate that P2X7 receptor-mediated Ca^2+^ influx is important for the activation of the metalloproteinase TACE and responsible for boosting TNF-α release, describing an additional pro-inflammatory signaling pathway modulated by P2X7 receptor in macrophages. Similarly, the release of TNF-α by P2X7 receptors upon Ca^2+^ influx has been also described in fibroblasts (22). TNF-α is a key cytokine implicated in apoptosis and inflammation, being its dysregulated production involved in rheumatoid arthritis or Crohn’s disease, where anti-TNF-α therapy is approved for clinical use (13, 14). Drug development targeting P2X7 receptor has been a pillar for different pharmaceutical companies in the search of novel anti-inflammatory molecules, being several drug-like P2X7 receptor antagonists in clinical trials, with phase IIb for rheumatoid arthritis and Crohn’s disease (5–7). Our study and others supports the idea that P2X7 receptor blocking could ameliorate different pro-inflammatory pathways without obtaining a complete immunoparalysis. Interestingly, we did not find any defect on the recruitment of inflammatory granulocytes into the peritoneum of P2X7 receptor deficient mice upon LPS or LPS + ATP injections, these data contrast with previous results where P2X7 deficiency reduced for example neutrophil infiltration upon cecal ligation and puncture mouse model of sepsis (36). However, in a different study, the cecal ligation and puncture resulted in a similar infiltration of neutrophils when compared to wild-type and P2X7 receptor knockout mice (37). These differences might be explained because bacterial-induced ATP release in the peritoneum stimulates a direct chemotaxis *via* P2Y receptors and an indirect infiltration *via* P2X7 receptor engaging the NLRP3 inflammasome and releasing IL-1β (38). In our model, the LPS challenge could induce the main recruitment of inflammatory cells and might mask the potential effect of further recruitment by a second inoculation of exogenous ATP *via* P2X7 receptor-dependent IL-1β release.

Meanwhile, P2X7 receptor-inducing IL-1β release has been widely studied, we report that IL-1β release goes from nothing to saturation at concentrations of ATP ≥3 mM, confirming that inflammasome activation follow an all-or-none activation step after P2X7 receptor activation (4, 39). In contrast, ATP-inducing TNF-α release was gradually enhanced from macrophages treated at increasing concentrations of ATP. Although ATP EC_50_ for mouse P2X7 receptor is ~900 μM (40, 41), the small concentrations of ATP-inducing TNF-α release from macrophages could activate different purinergic receptors (i.e., P2X4 or P2Ys) and induce low intracellular Ca^2+^ rise enough to activate MAPK and TACE (10, 28). In line, ATP stimulation after LPS priming on P2X7-deficient macrophages or after specific blocking of P2X7 receptor by A740003 or AZ10606120 results in a small and not significant increase of TNF-α release when compared to LPS alone treated macrophages, supporting the idea that other purinergic receptors could minimally contribute to this response. However, activation of P2X7 receptor leads to a robust increase of intracellular Ca^2+^ and a burst in the release of TNF-α. This phenomena is also observed when the elevation of intracellular Ca^2+^ by triggering P2X4 receptor increase prostaglandin E2 production in LPS-primed macrophages, and then, prostaglandin production is further increase when P2X7 receptor is fully activated at ATP concentrations ≥1 mM (10, 42). However, P2X4 signaling do not activate the NLRP3 inflammasome and, therefore, do not induce IL-1β release (10, 43). Our study also reveal that P2X7 receptor induces TNF-α release from the surface of the plasma membrane by activating the metalloproteinase TACE, being TACE activity tightly modulated by p38 MAPK (15). Our data suggest that P2X7 receptor controls TACE activity *via* p38 activation and is well described that MAPK activation is controlled by the rise of intracellular Ca^2+^ after P2X7 receptor stimulation (10, 44, 45). Furthermore, at low concentrations of LPS, MAPK signaling is not activated (46) and in these conditions, P2X7 receptor-induced p38 activation could be the trigger to increase TACE activity and induce TNF-α release.

TNF-α converting enzyme is a membrane-anchored metalloproteinase important for the shedding of different membrane-anchored cell-surface proteins. TACE substrates include TNF-α, IL-1 receptor type II, TNF-receptor, transforming growth factor-α, or l-selectin (47). P2X7 receptor has been previously found to induce l-selectin shedding *via* metalloproteinases (12, 48, 49), and our work suggests that TACE could be part of the metalloproteinase activity in charge of l-selectin shedding. Our study demonstrates that together with an increase of TNF-α release, P2X7 receptor stimulation also induces the release of TACE from the cell. We present a new mechanism to generate an extracellular form of TACE that is able to induce the shedding of TNF-α, and presumably other substrates as l-selectin, from neighbor cells that present a decreased or deficiency activity of TACE. Although this is the first report demonstrating a regulatory mechanism to induce an extracellular form of TACE in exosomes from macrophages, the identification of this metalloprotease in exosomes has been already reported in colon cancer (50). Elevated levels of TACE activity has been also described in the plasma and cerebrospinal fluid of Alzheimer’s patients, together with an increase of soluble TNF-receptors (51, 52). Therefore, extracellular TACE emerge as a marker for disease, and our results indicate that P2X7 receptor could be involved in maintaining extracellular active TACE.

In different cell types, purinergic signaling modulates *TNFA* gene expression and consequently the release of TNF-α. In microglia, P2X7 receptor activation induces TNF-α production by *de novo* gene expression after 24 h exposure to ATP in the absence of LPS (53). Also, in mononuclear cells, TNF-α gene expression induced by LPS or *Mycobacterium* has been shown to be affected by purinergic signaling (54, 55). In macrophages, the degradation of ATP to adenosine resulted in a reduction of TNF-α by A2A receptor activation (54). Stimulation of human whole blood cells with ATP, but not BzATP, reduced LPS-induced TNF-α production (55). Our study suggests that in LPS-primed macrophages, P2X7 receptor activation potentiate, in 20 min, the release of TNF-α without affecting gene expression by boosting its shedding from the plasma membrane, this mechanism is distinct to other reports showing how ATP modulates TNF-α gene expression (53–55). Although our study mainly investigate mouse macrophages, is in line with the fact that individuals carrying gain-of-function SNPs on *P2RX7* gene present an increase on TNF-α release in whole blood assays after LPS stimulation (56), and in human monocytes LPS treatment induces the release of ATP and activate P2X7 receptor using an autocrine/paracrine loop revealed by the activation of the NLRP3 inflammasome (57). Therefore, the release of TNF-α *via* P2X7 receptor activation could be relevant in pathophysiological conditions where, for example, the toll- and MAPK-signaling pathways are extenuated and the production of pro-inflammatory cytokines is dampened.

In conclusion, our work presents a mechanism to control TACE and TNF-α release from LPS-primed macrophages by P2X7 receptor activation. This mechanism is distinct from the activation of the NLRP3 inflammasome and suggests that multiple pro-inflammatory pathways are associated to P2X7 receptor, supporting the development of P2X7 receptor antagonists to use in chronic inflammatory diseases.

## Ethics Statement

Whole peripheral blood samples were collected from healthy donors upon approval of the Hospital Clinico Universitario Virgen de la Arrixaca’s Clinical Review Board. An informed consent was obtained from all donors enrolled in the study following the principles set out in the WMA Declaration of Helsinki. Animals were used in accordance with the Hospital Clinico Universitario Virgen Arrixaca animal experimentation guidelines, and the Spanish national (RD 1201/2005 and Law 32/2007) and EU (86/609/EEC and 2010/63/EU) legislation. Animal procedure was refined and approved by the Hospital Clinico Universitario Virgen Arrixaca animal experimentation committee and approved by the Servicio de Sanidad Animal, Dirección General de Ganadería y Pesca, Consejería de Agricultura y Agua Región de Murcia (Health Animal Service, Murcia Fishing, and Farming Council, reference C1310050308).

## Author Contributions

MB-C, AIG, CMM, CdT-M, and AB-M performed the experiments; MB-C, CdT-M, and PP analyzed the data and prepared the figures; LM-A provided human samples. CdT-M and PP wrote the paper. PP provided funding, conception, and overall supervision of this study.

## Conflict of Interest Statement

The authors declare that the research was conducted in the absence of any commercial or financial relationships that could be construed as a potential conflict of interest. The reviewer, MF, and handling editor declared their shared affiliation, and the handling editor states that the process nevertheless met the standards of a fair and objective review.
